# Tolerability of concurrent external beam radiotherapy and [^177^Lu]Lu-PSMA-617 for node-positive prostate cancer in treatment naïve patients, phase I study (PROQURE-I trial)

**DOI:** 10.1186/s12885-023-10725-5

**Published:** 2023-03-23

**Authors:** Esmée C. A. van der Sar, Arthur J. A. T. Braat, Jochem R. N. van der Voort- van Zyp, Betty S. van der Veen, Pim J. van Leeuwen, Daphne M. V. de Vries-Huizing, Jeroen M. A. Hendrikx, Marnix G. E. H. Lam, Wouter V. Vogel

**Affiliations:** 1 Department of Radiology and Nuclear Medicine, University Medical Center Utrecht, Utrecht, The Netherlands; 2 Department of Radiation Oncology, University Medical Center Utrecht, Utrecht, The Netherlands; 3 Department of Radiation Oncology, Netherlands Cancer Institute NKI-AVL, Amsterdam, The Netherlands; 4 Department of Urology, Netherlands Cancer Institute NKI-AVL, Amsterdam, The Netherlands; 5 Department Nuclear Medicine, Netherlands Cancer Institute NKI-AVL, Amsterdam, The Netherlands; 6 Department of Pharmacy & Pharmacology, The Netherlands Cancer Institute, Amsterdam, The Netherlands

**Keywords:** Lu-PSMA, Prostate cancer, Node-positive

## Abstract

**Background:**

Prostate cancer patients with locoregional lymph node disease at diagnosis (N1M0) still have a limited prognosis despite the improvements provided by aggressive curative intent multimodal locoregional external beam radiation therapy (EBRT) with systemic androgen deprivation therapy (ADT). Although some patients can be cured and the majority of patients have a long survival, the 5-year biochemical failure rate is currently 29–47%. [^177^Lu]Lu-PSMA-617 has shown impressive clinical and biochemical responses with low toxicity in salvage setting in metastatic castration-resistant prostate cancer. This study aims to explore the combination of standard EBRT and ADT complemented with a single administration of [^177^Lu]Lu-PSMA-617 in curative intent treatment for N1M0 prostate cancer. Hypothetically, this combined approach will enhance EBRT to better control macroscopic tumour localizations, and treat undetected microscopic disease locations inside and outside EBRT fields.

**Methods:**

The PROQURE-I study is a multicenter prospective phase I study investigating standard of care treatment (7 weeks EBRT and 3 years ADT) complemented with one concurrent cycle (three, six, or nine GBq) of systemic [^177^Lu]Lu-PSMA-617 administered in week two of EBRT. A maximum of 18 patients with PSMA-positive N1M0 prostate cancer will be included. The tolerability of adding [^177^Lu]Lu-PSMA-617 will be evaluated using a Bayesian Optimal Interval (BOIN) dose-escalation design. The primary objective is to determine the maximum tolerated dose (MTD) of a single cycle [^177^Lu]Lu-PSMA-617 when given concurrent with EBRT + ADT, defined as the occurrence of Common Terminology Criteria for Adverse Events (CTCAE) v 5.0 grade three or higher acute toxicity. Secondary objectives include: late toxicity at 6 months, dosimetric assessment, preliminary biochemical efficacy at 6 months, quality of life questionnaires, and pharmacokinetic modelling of [^177^Lu]Lu-PSMA-617.

**Discussion:**

This is the first prospective study to combine EBRT and ADT with [^177^Lu]Lu-PSMA-617 in treatment naïve men with N1M0 prostate cancer, and thereby explores the novel application of [^177^Lu]Lu-PSMA-617 in curative intent treatment. It is considered likely that this study will confirm tolerability as the combined toxicity of these treatments is expected to be limited. Increased efficacy is considered likely since both individual treatments have proven high anti-tumour effect as mono-treatments.

**Trial registration:**

ClinicalTrials, NCT05162573. Registered 7 October 2021.

**Supplementary Information:**

The online version contains supplementary material available at 10.1186/s12885-023-10725-5.

## Background

In the past, prostate cancer patients with nodal metastases were not considered for curative treatment, based on the assumption that cure was neither feasible nor beneficial, based on the hypothesis that patients with lymph node metastases are affected by a systemic disease. With the introduction of molecular imaging targeting the prostate-specific membrane antigen (PSMA), the number of patients diagnosed with primary node-positive prostate cancer without distant metastasis (staged N1M0) is increasing as PSMA PET/CT allows better detection of small nodal metastases and at the same time better exclusion of distant metastases (including nodal metastases in non-standard locations) with a high positive predictive value [1, 2]. These developments are leading to higher confidence that at least some of the current prostate cancer patients staged N1M0 may not yet have systemic disease, and therefore may potentially benefit from an aggressive multimodal treatment approach. Based on this assumption, more and more N1M0 patients are now offered high-dose locoregional external beam radiation therapy (EBRT) with a boost to all visible tumour lesions in the pelvic area combined with 2–3 years concurrent and adjuvant androgen deprivation therapy (ADT). This treatment is given with the intent to cure, or if this fails to at least provide maximum disease-free and overall survival [3].

Despite the improvements provided by aggressive multimodal locoregional EBRT with systemic long-term ADT, patients with N1M0 disease still have a suboptimal prognosis. Although the majority of patients have a long survival, curation is not always achieved and the 5-year (biochemical) failure rate is currently reported at 29–53% [4, 5]. In addition, patients receiving this treatment may experience a combination of toxicities, which may be persistent with potential impact on their remaining life. These disadvantages may be additional to surgical toxicity for patients who were staged N1 using lymph node dissection (LND) or sentinel node procedure (SNP) [6]. In combination, the current treatment with pelvic EBRT + ADT leads to many patients experiencing potentially significant reductions in quality of life (QoL), while still being confronted with recurrent and incurable disease within their remaining life. A new treatment strategy for these patients, which improves their chance of cure but also reduces toxicity and avoids reductions in QoL, is eagerly awaited.

Unfortunately, options are limited. Dose escalation of EBRT for prostate cancer is difficult due to toxicity in regional organs at risk. There are several new pharmaceutical options to intensify systemic ADT, but this has not been proven effective for primary treatment of N1M0 patients and it would likely increase long-term toxicity. The same is true for adding systemic chemotherapy, which was demonstrated to be not beneficial for low-volume metastatic patients and would also contribute to unwanted toxicity [7]. There are several directions to explore for further improvement of treatment. Firstly, current EBRT could be enhanced to better control macroscopic tumour in the prostate and detected involved nodes, secondly, undetected microscopic disease locations inside and outside EBRT fields need to be targeted with more efficacy, and, lastly, it could be beneficial to potentially shorten or obviate the long-term ADT with its associated poor QoL.

**Fig. 1:**
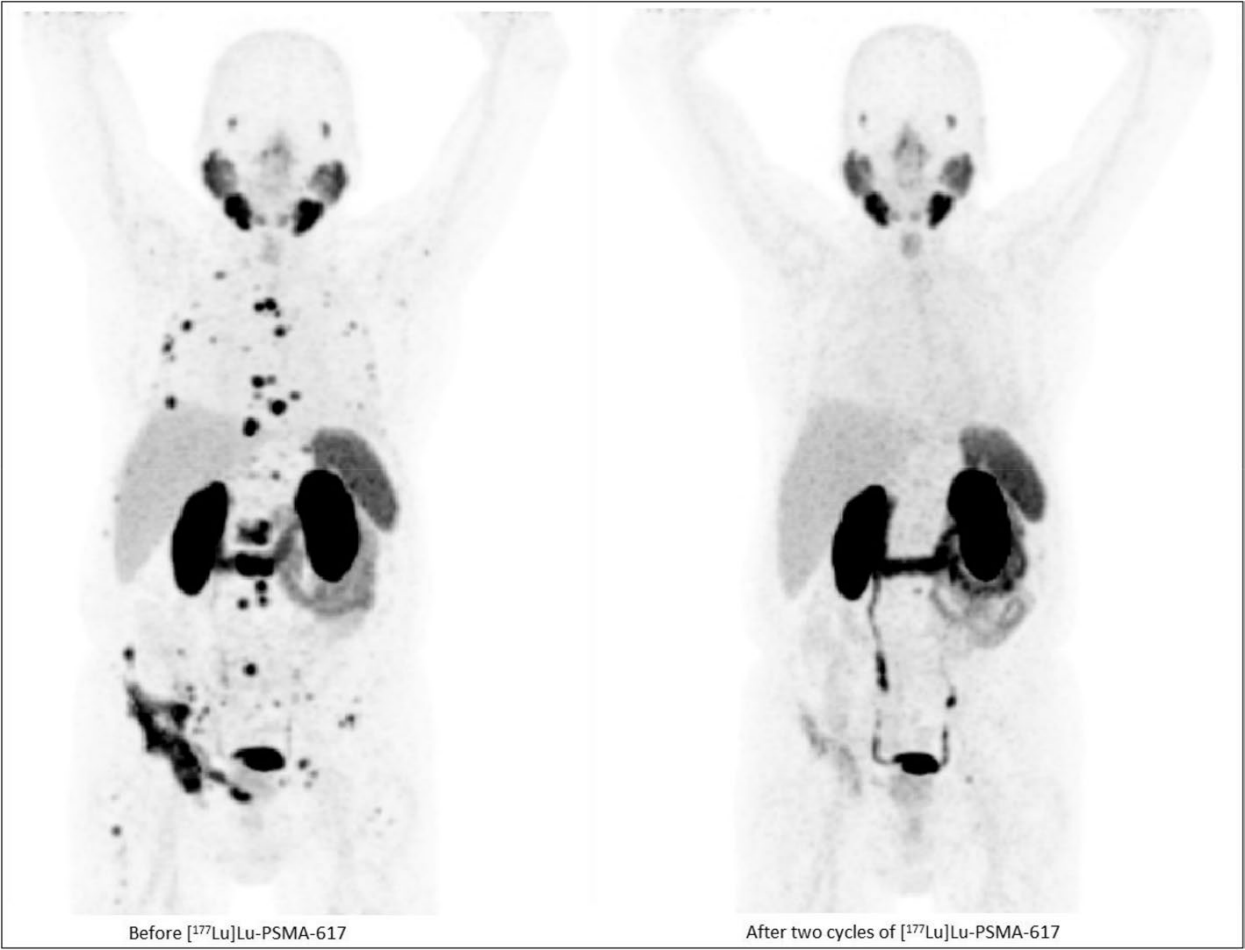
Example of response of [^177^Lu]Lu-PSMA-617 in a patient with metastatic castration-resistant prostate cancer. On the left side you see a [^68^ Ga]Ga-PSMA-11 PET maximal intensity projection (MIP) of a patient with metastatic prostate cancer in bone and lymph nodes. There is physiological accumulation in the salivary glands, kidneys, spleen, and to a much lower extent small and large bowel. On the right you also see a [^68^ Ga]Ga-PSMA-11 PET MIP from the same patient after two cycles of [^177^Lu]Lu-PSMA-617 which shows almost complete response

Radionuclide therapy using PSMA-ligand labelled with [^177^Lu]Lutetium-PSMA ([^177^Lu] (Lu-PSMA) may provide the desired improvements. Intravenous administration of [^177^Lu]Lu-PSMA leads to highly selective accumulation in tumour cells, and highly selective dose deposition within tumour locations. The short path length of the emitted electrons of about 1.5 mm contributes to low dose deposition in surrounding normal tissues, but still allows cross-fire and bystander effects in adjacent PSMA-negative tumour cells. This is notably not limited to known macroscopic (visible) lesions, that are detectable with e.g. a PET-scan, but it theoretically also includes undetected microscopic PSMA-positive tumour locations throughout the body. [^177^Lu]Lu-PSMA-617 therapy has already shown impressive clinical and biochemical responses with low toxicity in palliative setting in metastatic castration-resistant prostate cancer (mCRPC) (Fig. 1) [8, 9]. The recently published VISION study has shown complete biochemical response in 9.2% (17/184) mCRPC patients and PSA-decline of 50% or more in 41.8% (77/184) of the mCRPC patients receiving up to six cycles of [^177^Lu]Lu-PSMA-617 [8].

In case of high tracer accumulation in the primary tumour and nodal metastases, a similarly high dose delivery and good anti-tumour effect could be expected in the curative setting. In combination with EBRT and ADT, the concurrent and synergistic dose delivery could enhance EBRT to better control macroscopic tumour in the prostate and detected involved nodes, and it could also treat undetected microscopic disease locations inside and outside EBRT fields.

This study aims to explore the tolerability of the combination of standard EBRT and ADT complemented with a single administration of [^177^Lu]Lu-PSMA-617, for patients who are treated with curative intent for N1M0 prostate cancer.

## Method

### Study design and subjects

**Fig. 2:**
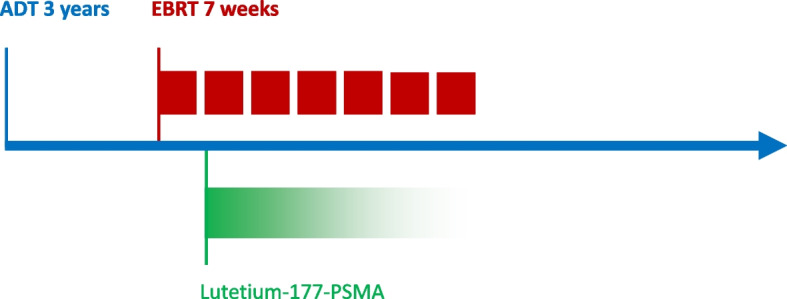
Study design. At the start of the treatment patients receive adjuvant hormone therapy (ADT) at most one month prior to the start of external beam radiation therapy (EBRT) and will continue during EBRT. Adjuvant hormone therapy is then continued as adjuvant treatment up to the advised total duration of 3 years (provided that its toxicity remains acceptable). In week two of EBRT, patients also receive one concurrent cycle of systemic [^177^Lu]Lu-PSMA-617 (Lu-PSMA) as part of the study procedure

The PROQURE-I study is a national multicenter prospective phase I dose-escalation study investigating the addition of one cycle of systemic [^177^Lu]Lu-PSMA-617 (Pluvicto™, Advanced Accelerators Applications) concurrent with standard of care treatment (7 weeks EBRT and 3 years ADT), in treatment naïve patients with PSMA-positive N1M0 prostate cancer (see Fig. 2). Detailed inclusion and exclusion criteria of the subjects are listed in Table 1.

**Table 1:** Inclusion and exclusion criteria

Inclusion criteria	Exclusion criteria
Histologically proven prostate cancer	Inability to comply to study procedures
cT2-4, partly determined by MRI	Inability to adhere to radiation safety measures in hospital or at home
N1, determined by LND/SNP and/or PSMA PET/CT	Inability to undergo the required biodistribution scans
iM0, determined by PSMA PET/CT	Prior or current malignant disease with potential impact on treatment outcome or survival
Accepted for curative intent treatment with EBRT of the prostate and regional nodes + three years ADT	Prior treatment with EBRT
Visually PSMA-positive primary tumour and nodes, largest lesion > average liver accumulation	Prior treatment with ADT, already initiated > one month before the start of EBRT
WHO performance score 0–1	Prior treatment with radionuclide therapies, [^177^Lu]Lu-PSMA-617 or other
Age > 18 years	Reduced bone marrow reserve (Hb < 6 mmol/L, Leukocytes < 2.5 10E9/L, or Platelets < 100 10E9/L not older than one month before start of EBRT)
For patients who have partners of childbearing potential: Willingness to use a method of birth control with adequate barrier protection during the study and for six months after the study drug administration	Reduced renal function (GFR < 60 ml/min/1,73m^2^ not older than one month before start of EBRT)
Signed written informed consent	Reduced salivary gland function (history of prior salivary gland disease)
	Urinary problems requiring pre-treatment with ADT^a^

*ADT* Androgen deprivation therapy, *CT* Computed Tomography, *EBRT* External beam radiation therapy, *GRF* Glomerular filtration rate, *Hb* Hemoglobulin, *LND* Lymph node dissection, *Lu-PSMA* Lutetium PSMA, *MRI* Magnetic resonance imaging, *PET* Positron emission tomography, *PSMA* Prostate specific membrane antigen, *SNP* Single node procedure, *WHO* World health organization

^a^to avoid diminished PSMA- expression or escalating radiation safety issues with radioactive urine at the time of [^177^Lu]Lu-PSMA-617

### Hypothesis

The hypothesis of this phase I study is that combining EBRT and ADT (in accordance to current standard of care) with one cycle of systemic [^177^Lu]Lu-PSMA-617 delivered in week two of EBRT, in treatment naïve patients with PSMA-positive N1M0 prostate cancer will not induce significant additional toxicity. It is important to exclude significant additional toxicity, because this could potentially lead to interruption of the standard curative intent treatment. To evaluate toxicity of a multimodal treatment, the toxicities of the separate components need to be considered.

The toxicity profile of ADT has been reported extensively, and includes e.g. fatigue, sexual dysfunction, gynecomastia, flushes and depression [10]. Beyond these acute issues, there is increasing awareness of the long-term effects. ADT leads to adverse changes in body weight, body composition, insulin resistance and serum cholesterols, together known as the “metabolic syndrome”, with changes already observed after only 12–24 weeks and largely persistent after the end of treatment [11].

The acute toxicity of EBRT of the prostate and pelvic nodes with concurrent ADT has also been reported extensively, for example in the PIVOTAL study in 2018 [12]. These data illustrate that grade 1–2 acute toxicities according to Radiation Therapy Oncology Group (RTOG) criteria are common, occurring in up to 30% for the gastrointestinal tract and up to 40% for the bladder, with their peak incidence at the end of EBRT. However, grade three acute toxicities are rare, below five % in all treatment weeks for all evaluated parameters. The late toxicity of pelvic EBRT was also reported in the same study [12], which reports that occurrence of late grade one toxicities according to CTCAE criteria are common, occurring in up to about 70% for the gastrointestinal tract as well as the bladder. Late grade two toxicities are rare, below 20% up to 2 years for the gastrointestinal tract and below 30% up to 2 years for the bladder. CTCAE grade one toxicities increase during at least 2 years after treatment, and their incidence at 6 months should be considered a relatively poor indicator. Whilst grade two toxicities show much less increase over time, and their incidence at 6 months is a reasonable indicator for toxicity later on. Finally, late grade three toxicities hardly occurred (< 5%).

[^177^Lu]Lu-PSMA-617 is considered a low toxicity treatment. Renal clearance of [^177^Lu]Lu-PSMA-617 from the circulation occurs rapidly, stimulated by standard good hydration, thus blood concentrations are low. Dosimetry data have indicated that binding occurs in PSMA-expressing normal tissues (besides tumours), which mainly include the salivary glands, kidneys, and to a much lower extent small and large bowel [13]. A study using six cycles of 6–9 GBq [^177^Lu]Lu-PSMA-617 reported grade one xerostomia in 87%, grade 1–2 transient nausea in 50%, grade 1–2 fatigue in 50%, and grade 3–4 thrombocytopenia in 13% of patients [14]. As expected, studies that applied only one or two cycles [^177^Lu]Lu-PSMA-617 reported much lower toxicity rates; xerostomia (8.7%), mild nausea (12.5%), fatigue (17.4%), no CTCAE grade 2–3 thrombocytopenia, and no grade four adverse events [9].

Based on these patterns we hypothesize that tolerability of EBRT and ADT will not be affected by adding one administration of up to nine GBq [^177^Lu]Lu-PSMA-617. The toxicity of ADT is considered to be largely independent of ionizing radiation, and is not expected to change in the scope of combining EBRT with [^177^Lu]Lu-PSMA-617. The dose distributions from EBRT and [^177^Lu]Lu-PSMA-617 will have a desired overlap in tumour locations in the pelvic area, but they have no significant overlap in the dose delivered to organs at risk and there is no overlap in their toxicity profiles. Most importantly, EBRT to the pelvic area does not involve any dose to the kidneys or the salivary glands. Vice versa, [^177^Lu]Lu-PSMA-617 does not have known bladder toxicity, and no gastrointestinal toxicity beyond transient nausea after infusion. Concurrent dose delivery from EBRT and unbound [^177^Lu]Lu-PSMA-617, for example to the bladder wall (from radioactive urine passing through) and to perfused organs in the pelvic area, can be minimized by administering the radiopharmaceutical shortly after an EBRT fraction to allow about 20 h to clear the urological tract and circulation before the next EBRT fraction is delivered.

Based on these considerations, the risk of significant extra toxicity due to the addition of one cycle of [^177^Lu]Lu-PSMA-617 concurrent with EBRT and ADT is considered limited, including for higher dose levels. The occurrence of significant additional toxicity can be evaluated by monitoring (treatment related) acute CTCAE v. 5.0 grade three or higher toxicity of any type during treatment, and late CTCAE v. 5.0 grade three or higher toxicity of any type at 6 months after treatment.

### Primary objectives

To determine the maximum tolerated dose (MTD) of one cycle [^177^Lu]Lu-PSMA-617 (three, six, or nine GBq) when administered in combination with standard EBRT and ADT. MTD will be determined according to the Bayesian Optimal Interval (BOIN)-design (see section 2: Dose escalation) [15]. Dose-limiting toxicity (DLT) is defined as an acute toxicity, occurring from the start of EBRT up to 3 months after completion of EBRT, according to CTCAE v. 5.0 grade three or higher of any type. As CTCAE grade three or higher toxicities are very limited in EBRT and ADT combination therapy, this is considered to be a suitable endpoint for tolerability in this study (see hypothesis above). Relation of acute CTCAE grade three toxicity or higher to the study treatment is determined by the principal investigators, and will be verified by a safety committee prior to escalation to a higher dose level.

### Secondary objectives

Five secondary objectives have been defined: first, late toxicity CTCAE v 5.0 grade three or higher will be evaluated at 6 months post-treatment. Second, dosimetric efficacy will be assessed using the summed EQD2 dose to screen for superiority of delivered dose to tumour (and organs at risk), as compared to EBRT alone. Third, anti-tumour efficacy will be assessed using the prostate-specific antigen (PSA) response level. This parameter cannot be used to detect improved anti-tumour effect for the experimental combined treatment, but it can be used to detect a (highly unlikely) reduced anti-tumour effect. Fourth, the feasibility of QoL evaluation by using QLQ-C30 and prostate-specific QLQ-PR25 questionnaires for this specific patient group will be determined. Lastly, pharmacokinetic modelling will be used to further explore the selected dose levels and the kinetics of [^177^Lu]Lu-PSMA-617 as compared to monotherapy. Sequential blood samples before and after treatment will be combined with three time point SPECT/CT and planar whole-body biodistribution imaging to generate a pharmacokinetic model for [^177^Lu]Lu-PSMA-617 in the presence of continued fractionated EBRT.

### Investigational medicinal product

The radiopharmaceutical [^177^Lu]Lu-PSMA-617 (Pluvicto™, Advanced Accelerators Applications) is comprised of the isotope lutetium-177 ([^177^Lu]Lu) linked to a small molecule targeting the PSMA receptor. The beta-emitting [^177^Lu]Lu has a physical half-life of 6.6 days, medium-energy beta-emission (490 keV), and a maximum electron tissue path length of about 1.5 mm [16]. [^177^Lu]Lu-PSMA-617 is considered a low toxicity treatment based on previous prospective clinical trials (see also section hypothesis) [8, 14].

### Estimated total dose

The respective doses from the two different modalities need to be considered to provide an estimation of the total delivered dose.

First, EBRT physical doses can be converted to doses equivalent for two Gy fraction using the α/β (alpha/beta, fractionation sensitivity) factor. The α/β for prostate cancer has been estimated from very low (1.2) to medium (4.9) in various studies [17, 18]. In the scope of this study an intermediate α/β of three is assumed. This leads to radiobiologically equivalent doses (as delivered in two Gy fractions, EQD2_a/b3_) of 80 Gy to the prostate, 56 Gy to macroscopic nodal metastases, and 47 Gy to microscopic nodal metastases in the elective fields (Table 2).

**Table 2:** Total dose for concurrent EBRT with [^177^Lu]Lu-PSMA-617, estimated for various tumour locations and for various [^177^Lu]Lu-PSMA activity levels

Tumour dose in Gy (EQD2)	EBRT	3 GBq [^177^Lu]Lu-PSMA-617			6 GBq [^177^Lu]Lu-PSMA-617			9 GBq [^177^Lu]Lu-PSMA-617		
**Tumour location**	**Alone**	**Alone**	**Sum**	** + 6%**	**Alone**	**Sum**	** + 6%**	**Alone**	**Sum**	** + 6%**
Primary tumour	80	6	86	**91**	12	92	**98**	18	98	**104**
Macroscopic nodes in boost field	56	9	65	**69**	18	74	**78**	27	83	**88**
Microscopic nodes in elective field	47		56	**59**		65	**69**		74	**78**
Microscopic lesions outside fields	0		9	**9**		18	**18**		27	**27**

Values are expressed in Gy (EQD2), calculated with α/β = 3 for (metastatic) prostate cancer. The column marked + 6% includes an assumed synergistic effect for tumour locations that receive dose from the combined treatments

*Lu-PSMA* Lutetium PSMA, *PSMA* Prostate specific membrane antigen

Second, [^177^Lu]Lu-PSMA-617 delivers dose to tumour with a low dose-rate over a period of ~ 3 weeks, which has been estimated at about 2.5–3.5 Gy per administered GBq depending on the tumour location [19]. For primary tumours the tracer accumulation may be more variable, and is generally lower than metastatic locations that typically consist of selected poorly differentiated and highly PSMA-expressing tumour cells. For this study, which selectively includes patients with relatively high PSMA-expression in all known lesions, we will assume a delivered dose of two Gy/GBq to the primary tumour, and three Gy/GBq for all other tumour locations.

On top of added dose, concurrent dose delivery by EBRT and [^177^Lu]Lu-PSMA-617 can provide synergetic effects that have been suggested conservatively in the range of 6% for low α/β tumours like prostate cancer [20]. Based on these considerations, we estimate that administration of one single dosage of [^177^Lu]Lu-PSMA-617 during the second week of EBRT would translate to the total doses listed in Table 2.

### Recruitment

Patients will be identified at multidisciplinary uro-oncology tumour boards, at the time of confirmed PSMA-positive prostate cancer, staged N1M0, and referred for EBRT and ADT. When patients accept EBRT and ADT, they are subsequently informed about this study. Patients will receive oral and written information from their treating physician, and will be given at least 72 h to consider participation in the study. Patients who agree to participate will sign the informed consent form. Patients who decline participation will receive standard of care treatment (EBRT and ADT).

### Time schedule

Recruitment has started in January 2022 and is expected to complete before July 2023.

### Withdrawal of individual patients

Subjects can leave the study at any time for any reason if they wish to do so without any consequences. The investigator can decide to withdraw a subject from the study for urgent medical reasons. Patients withdrawn from the study will continue to receive the standard of care treatment and follow-up as deemed appropriate by the treating physician and according to current clinical protocol. This includes evaluation and management of relevant toxicities. No additional follow-up is required.

### Dose escalation

This study will use a Bayesian Optimal Interval design (BOIN design) with a target dose-limiting toxicity (DLT) rate of 30% and three pre-specified activity levels [15]. The three pre-specified activity levels of the [^177^Lu]Lu-PSMA-617 administration are: level one: three GBq, level two: six GBq and level three: nine GBq. Planned enrolment of a maximum of 18 patients or fewer (depending on the observed rate of DLTs) will be used to evaluate safety and tolerability. The DLT rate is calculated as the number of patients experiencing a DLT during the current dose divided by the total number of patients treated at the current dose.

To accelerate dose escalation, based on the knowledge of limited toxicity with a single administration [9], the trial starts with a cohort size of one patient per activity level. A cohort is expanded to three patients if the first DLT is observed. If no DLT is observed at activity levels one and two, the cohort size at the highest activity level (level three) will also be expanded to three patients. If no DLT is observed at all, the trial is terminated at the last activity level if the upper limit of the 95% binomial confidence interval (CI) is below 0.5 and the last activity (level three: nine GBq) is then selected as the MTD. This means that when zero out of six patients have a DLT at activity level three, the trial is terminated, because the 95% CI then ranges from zero to 0.46.

If a DLT is observed, the decision to expand the cohort and retain the current activity, to de-escalate, to escalate or to stop the trial is based on the pre-specified activity escalation and de-escalation boundaries for a DLT rate of 30% (λe = 0.236, λd = 0.358) and maximum sample size according to the BOIN design principles. The resulting decision rules are presented in Table 3. In addition to these decision rules, the following rules for safety are embedded. In level one, the trial will be stopped for safety if 2/3 or 3/3 patients experience a DLT. In activity level two and three, the activity will be eliminated according to the rules specified in the last row of Table 3. Elimination of a activity indicates that the current activity and higher activities will not be investigated anymore and the trial continues on a lower activity level.

**Table 3:** Escalation, de-escalation, elimination and stopping boundaries for each level depending on the number of patients with DLT

Action	**The number of patients treated at each dose**																	
	**1**	**2**	**3**	**4**	**5**	**6**	**7**	**8**	**9**	**10**	**11**	**12**	**13**	**14**	**15**	**16**	**17**	**18**
Level 1:																		
Stop the trial if # of DLT > =	NA	NA	2	3	3	4	4	4	5	5	6	6	6	7	7	7	8	8
Level 2 and 3:																		
Escalate if # of DLT < =	0	0	0	0	1	1	1	1	2	2	2	2	3	3	3	3	4	4
De-escalate if # of DLT > =	1	1	2	2	2	3	3	3	4	4	4	5	5	6	6	6	7	7
Eliminate dose if # of DLT > =	NA	NA	3	4	5	5	6	6	7	7	7	8	8	9	9	10	10	11

*DLT* Dose limiting toxicity, *NA* Not applicable, # number

### Temporary inclusion stop

After the last inclusion of each cohort, the inclusion will temporarily freeze until 1 month after the last EBRT of that cohort has been given. The occurrence of acute CTCAE grade three toxicity from EBRT and ADT is known to be limited (see also section 2: Hypothesis). When a patient has not demonstrated any grade three or higher acute toxicity well into the recovery phase at 1 month after the last EBRT fraction, the chance of newly developing grade three acute toxicity is extremely small and the primary endpoint will be considered negative for the purpose of dose escalation for subsequent patients. This means that, independent from the EBRT scheme, there will also be at least 2 months follow-up after the [^177^Lu]Lu-PSMA-617 administration for each patient.

### Monitoring and data management

The study will be risk based monitored according to International council on good clinical practice (ICH GCP) by an independent Clinical Research Monitor, including source data verification. Although toxicity is expected to be low, this phase I protocol will be considered as a high-risk study.

Central data management will be performed at the Data Center of the Netherlands Cancer Institute. All collected patient data in this trial will be coded and used in a pseudonymized manner. Registration will be performed using the ALEA® (FormsVision BV) registration package (FormsVision BV). All data that is relevant for the study will be collected using electronic Case Report Forms (eCRFs) in ALEA. Checks are incorporated into the eCRF system and data from all patients is centrally checked at the Data Center. When persistent irregularities or protocol violations are detected, the Data Center will inform the local investigator (and Principal Investigators) and queries will be sent to the local Data Manager. The completed eCRFs will be reviewed, signed and dated by the principal investigator or sub‐investigator.

In accordance with the Dutch regulations, the investigator will retain all pertinent information for a period of 25 years from study completion. Trial data is only accessible to the study physician, principal investigator and external data monitor. The handling of personal data will comply with the General Data Protection Regulation (GDPR) (in Dutch: AVG, Algemene Verordening Gegevensbescherming).

### Ethical considerations

The study has been approved by the Ethics Committee of the Netherlands Cancer Institute. This study is conducted in agreement with either the Declaration of Helsinki or the laws and regulations of the Netherlands, whichever provides the greatest protection of the patient. The study will be conducted according to the ICH Harmonized Tripartite Guideline for Good Clinical Practice.

### Fundings

This phase-I study received no external funding. The study is supported by Advanced Accelerator Applications International S.A. Geneva, Switzerland, with supply of [^177^Lu]Lu-PSMA-617 free of charge and an unrestricted research grant to support data management and monitoring.

### Treatment

#### Standard treatment scheme

**Fig. 3:**
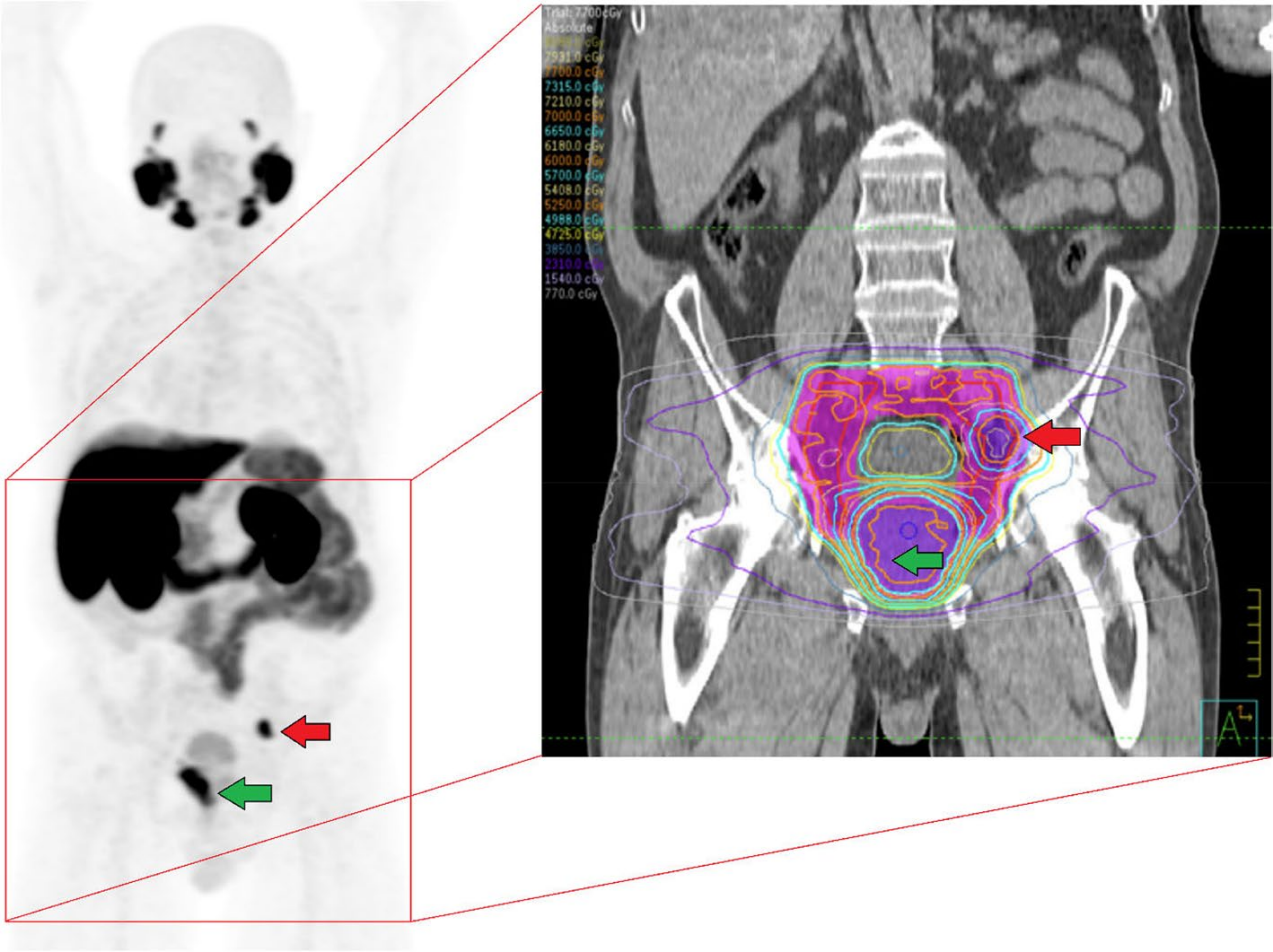
Example radiation field. Example of a radiation field in a patient with primary prostate cancer (indicated by a green arrow) and one lymph node metastasis in the left pelvic area (indicated by a red arrow) who is eligible for external beam radiation therapy (EBRT) and participation in the study. The delivered physical EBRT dose is 70–77 Gy to the prostate (purple area with green arrow), 60 Gy to macroscopic lymph node metastases (purple area with red arrow), and 52.5 Gy to the RTOG-based elective pelvic nodal fields expanded to include all detected nodal metastases (pink area)

Included patients will receive standard of care EBRT and ADT with curative intent according to current clinical guidelines. EBRT involves 35 fractions intensity modulated radiotherapy (IMRT), 5 days per week over 7 weeks, delivering physical doses of 70–77 Gy to the prostate (and seminal vesicles if applicable), 60–63.35 Gy to macroscopic lymph node metastases, and 52.5–56.35 Gy to RTOG-based elective pelvic nodal fields expanded to include all detected nodal metastases (Fig. 3). ADT consists of 3-monthly subcutaneous depots of 10.8 mg gosereline or equivalent treatment, with 4 weeks bicalutamide 50 mg orally from 2 weeks before till 2 weeks after the first administration. ADT is standard initiated at most 1 month prior to the start of EBRT (counting from the start of bicalutamide), is continued during EBRT, and is then continued as adjuvant treatment up to the advised total duration of 3 years (provided that its toxicity remains acceptable).

#### [^177^Lu]Lu-PSMA-617

In this study, the standard of care (EBRT and ADT) is complemented with [^177^Lu]Lu-PSMA-617, intravenously as a slow bolus. About one hour prior to administration of [^177^Lu]Lu-PSMA-617, patients will receive prophylactic anti-emetic medication (granisetron one mg per os, or equivalent medication).

After administration, patients will remain in isolation according to national guidelines for radiation protection, for at least six hours. During this period, patients will be monitored for acute toxicity (infusion reactions). Afterwards, patients are discharged and need to adhere to radiation safety measures according to current local and national guidelines, at home and during continued treatment procedures specifically including the remaining EBRT fractions.

#### Imaging

Biodistribution whole body planar and SPECT/CT imaging from the pelvis will be acquired at four, 24, and 120–168 h after administration of [^177^Lu]Lu-PSMA-617 for our secondary endpoint: absorbed dose in the target lesions and in organs at risk. Imaging will be performed on Symbia T or Intevo Bold systems (Siemens GmbH, Erlangen, Germany), equipped with a Medium Energy General Purpose collimator. Total body scintigraphy will be acquired with both heads at 15 cm/min, 256 × 1024 matrix and energy windows around the main photopeaks: 208 keV ± 10% and 113 keV ± 10%. SPECT/CT of the pelvis will be acquired with triple energy windows (TEW) around both photopeaks: 208 keV ± 10% with an adjacent upper and lower scatter window of 16% width of the 208 keV peak, and 113 keV ± 10% with an adjacent upper and lower scatter window of 24% and 12% width of the 113 keV peak, respectively. SPECT acquisition parameters are non-circular, continuous rotations of both heads with 64 views of 20 s/view per head. The SPECT image matrix size is 128 × 128 with 4.8 mm cubic voxels. SPECT reconstruction includes attenuation and scatter corrected 3DOSEM (FLASH3D) with six iterations and eight subsets with five mm Gaussian post-reconstruction filtering. Local cross calibration between the gamma camera and dose calibrator was performed to enable quantitative measurements.

#### Escape medication

Standard of care escape medication for EBRT and ADT includes e.g. tamsulosine or solifenacine (for radiation cystitis with increased frequency), loperamide or laxatives (for radiation enteritis or obstipation), dexamethasone (for inflammation or general deteriorating condition), or tamoxifen (for gynecomastia). Related to the investigational medicinal product [^177^Lu]Lu-PSMA-617, patients may receive additional anti-emetic medication (granisetron one mg or equivalent) if needed.

#### Follow-up

After treatment, patients will already be followed at the outpatient clinic according to standard of care of EBRT and ADT. The first visits will take place at three and 6 months (see Table S1). During these visits patients will undergo a clinical evaluation, laboratory testing (i.e. PSA-level), and will be checked for (serious) adverse events.

Two additional follow-up procedures will take place for this phase I study: first, during the third and 6 months visits patients are also asked to fill out EORTC questionnaires (QLQ-C30 and QLQ-PR25) and second, during the first 2 months of follow-up (recovery period) patients will be contacted twice to check for (serious) adverse events (see Table S1).

After 6 months of follow-up the study will end.

## Discussion

Current treatment (EBRT and ADT) for patients diagnosed with primary node-positive prostate cancer without distant metastasis comes with significant toxicity and reductions in QoL, while still being confronted with recurrent and incurable disease within their remaining life. In the current study, the tolerability of innovative combined modality treatment strategy will be explored (standard of care; EBRT of the prostate and regional pelvic nodes combined with 2–3 years ADT) complemented with systemic [^177^Lu]Lu-PSMA-617) for patients with N1M0 prostate cancer. Which may lead to a better tumour control while potentially reducing or obviating ADT and its associated toxicity for future patients.

The risk of significant extra toxicity due to the addition of one cycle of [^177^Lu]Lu-PSMA-617 concurrent with EBRT and ADT is considered limited, including for higher activity levels. Still, the dose distributions from EBRT and [^177^Lu]Lu-PSMA-617 will have a (desired) overlap in tumour locations in the pelvic area, but there is virtually no overlap in the dose delivered to organs at risk or in their respective toxicity profiles. However, for a limited number of target areas that receive concurrent doses from EBRT and [^177^Lu]Lu-PSMA-617, additional toxicity should be considered.

The PSMA-positive primary tumour in the prostate may receive very high combined doses from EBRT with [^177^Lu]Lu-PSMA-617. Normal prostate tissue does not show membrane expression of the PSMA epitope [21], but depending on the [^177^Lu]Lu-PSMA-617 activity level, an estimated dose of 94–113 Gy EQD2 may be delivered to index tumour lesions. Although most tumours originate in the peripheral zone of the prostate, some tumours may be directly adjacent to (or even invading) the urethra, bladder wall or rectal wall. Even with the extremely sharp drop-off in dose from [^177^Lu]Lu-PSMA-617, estimated at 10% at 0.3 mm from the tumour, there may be some additional local toxicity to these normal tissues [22]. This is not considered a problem, based on historic experience with local dose escalation strategies. In the recently published FLAME trial, patients with intermediate and high-risk prostate cancer received a physical EBRT dose of 95 Gy in 35 fractions (108 Gy EQD2 at α/β = 3) to MRI-based index lesions in the prostate, with no increase in genito-urological (GU) and gastro-intestinal (GI) toxicity compared to the standard treatment [23]. Using modern hypofractionated stereotactic body radiotherapy (SBRT) of the prostate, a dose of 45 Gy in five fractions of nine Gy (108 Gy EQD2 at α/β = 3) can be delivered safely even with a very short overall treatment time of just 5 days [24]. In the ASCENDE-RT trial patients with intermediate and high-risk prostate cancer received an EBRT dose of 46 Gy EQD2 complemented with a very high dose of 115 Gy local low-dose-rate brachytherapy; this extreme total dose resulted in a higher incidence of acute and late GU morbidity (grade three genito-urological events 18.4 versus 5.2%) and a non-significant trend for worse GI morbidity (grade three gastro-intestinal events 8.1 versus 3.2%) [25]. The current trial with EBRT and [^177^Lu]Lu-PSMA-617 delivers substantially lower total doses to index lesions in the prostate, even in the highest activity level. In the (unlikely) situation that increased local toxicity occurs, it should be identified by the selected toxicity criteria, leading to the identification of non-tolerability at the applied activity level.

The total dose delivered to tumour deposits in regional involved nodes is higher than any preceding study, up to 88 Gy EQD2 for macroscopic metastasis in the highest activity level. However, due to the sharp drop-off in the dose from [^177^Lu]Lu-PSMA-617, this is not expected to result in additional dose delivery or toxicity in surrounding normal tissues.

The toxicity of ADT is considered to be largely independent of ionizing radiation, and is not expected to change in the scope of combining EBRT with [^177^Lu]Lu-PSMA-617.

The combination of locoregional EBRT and systemic radioligand therapy is not new [26]. Combining EBRT with [^177^Lu]Lu has been demonstrated before in a single clinical study, although in a sequential approach and using a different molecular tracer. Patients with inoperable meningioma received a single cycle/activity of 7.4 GBq [^177^Lu]Lu-octreotate, followed by 30 × 2 Gy EBRT that was initiated 2–9 days later. In this setup, a limited part of the dose delivery from [^177^Lu]Lu-octreotate overlapped with EBRT. The study reported good clinical responses with no CTCAE grade three or higher toxicity, and no need to adapt the dose or distribution of EBRT except in cases where the optic nerve was positioned directly adjacent to tumour [22]. Another example in literature (using a different beta-emitting radionuclide) is EBRT concurrent with p-[^131^I]iodo-L-phenylalanine (IPA-131) for malignant gliomas [27], which was considered safe and potentially effective. This latter combination is now actively investigated in an international phase I study, with the hypothesis of synergistic effects based on the same radiobiological considerations (ClinicalTrials.gov Identifier: NCT03849105).

The benefit of combining EBRT with [^177^Lu]Lu-PSMA-617 for prostate cancer is potentially much larger when compared to these prior efforts because of multiple factors: first, The tumour-background ratio of PSMA-ligands is of the same magnitude as octreotate, and higher than phenylalanine. Second, both EBRT and [^177^Lu]Lu-PSMA-617 already have proven significant effect as separate treatments. Third, N + M0 prostate cancer involves patients in a generally better clinical condition and with a potentially curable disease, compared to the palliative situation of recurrent treatment refractory meningioma or recurrent glioblastoma. Fourth, the total number of patients with N1M0 prostate cancer requiring treatment is much higher, providing a larger cohort for scientific evaluation and a larger total benefit to society.

Since the expected anti-tumour effect of combining multiple treatments that each have individual proven anti-tumour effect is high, and the combined toxicity of these treatments is expected to be limited, it is likely that this study will result in successful dose-finding with confirmed tolerability and improved dosimetric efficacy. Subsequent studies will prove the future potential benefit of concurrent EBRT with [^177^Lu]Lu-PSMA-617 for patients with N1M0 prostate cancer. Firstly, better tumour control leads to longer disease-free survival. Secondly, better tumour control could obviate the need for the currently applied 2–3 years ADT, or reduce its duration, with significant reductions in toxicity. Thirdly, better tumour control could allow future dose reductions for EBRT, thereby allowing for fewer fractions, lower toxicity, and better tolerability. In combination, this could improve overall survival as well as the quality of life of treated patients, while reducing the total treatment time as well as costs.

Potential further improvements to the treatment strategy may be identified in due time. Anticipated options include: First, intra-arterial administration of [^177^Lu]Lu-PSMA-617, to achieve a higher tumour accumulation by boosting the first-pass effect. Second, the addition of a second cycle of [^177^Lu]Lu-PSMA-617 to further improve tumour control. Third, hypofractionation of pelvic EBRT from seven to five weeks to reach complete overlap with [^177^Lu]Lu-PSMA-617 while reducing the overall treatment time and costs, which has already been demonstrate a safe strategy as a standalone treatment [28]. These options will require careful interpretation of results from this study, as well of new evidence in scientific literature.

There are other groups that could benefit from similar treatment intensification with [^177^Lu]Lu-PSMA-617. The most comparable group involves patients with pelvic nodal recurrence after prostatectomy, who are increasingly treated with pelvic EBRT (with or without the prostatic fossa) and 2–3 years ADT [29]. A second related group involves patients with primary or recurrent oligometastatic prostate cancer, where all macroscopic disease receives local ablative radiotherapy but assumed microscopic metastasis could benefit from additional dose. A third group involves localized high-risk prostate cancer, clinically N0 but with significant risk of missed microscopic metastatic disease (e.g. according to nomograms). Based on the results of this study, prospective research could be expanded to include these patients.

Currently, there are three other studies registered that are also investigating Lu-PSMA in hormone naïve prostate cancer patients. Firsty, PSMAddition (NCT04720157), an international, multicenter, open-label, randomized, phase III study investigating [^177^Lu]Lu-PSMA-617 combining with ADT in hormone naïve prostate cancer patients in comparison to standard of care. The study opened in 2021 and recruiting is ongoing. Secondly, Bullseye 2 (NCT04443062), a multicenter, randomized, open-label, phase II study, investigating [^177^Lu]Lu-PSMA-617 in men with recurrence prostate cancer who are eligible for ADT. The study opened in 2020 and recruiting is ongoing. Third, LUNAR (NCT05496959) [30], singlecenter, open-label, randomized, phase II study, investigating neoadjuvant ^177^Lu-PNT2002 plus SBRT versus SBRT alone in men with oligorecurrent hormone sensitive prostate cancer. This study opened in 2022 and is also still recruiting.

This study protocol has some limitations: Firstly, the BOIN-design is chosen for more flexibility in: the chosen target toxicity rate and cohort size and has a better performance in comparison to 3 + 3 design [15]. However the downside is that a tighter de-escalation boundary to impose a higher safety requirement and decreasing the risk of overdosing, may also reduce the percentage of correct selection and the number of patients allocated to the MTD.

Secondly, the choice to start with three GBq was based on earlier safety and efficacy studies, where they used a administered activity of six GBq [^177^Lu]Lu-PSMA-617 for multiple times in one single patient [31, 32]. Because this study is a phase I study combining EBRT with one cycle of [^177^Lu]Lu-PSMA-617, we chose to start with half of the activity (three GBq), out of precaution. The use of nine GBq has been proven to be safe [33], thus a starting dose of three GBq to a maximum of nine GBq is well grounded in our opinion. However, the best dose to administer may be higher, lower or somewhere in between the selected activity of three, six and nine GBq. Lastly, in this phase I study patients only get one cycle of [^177^Lu]Lu-PSMA-617 to evaluate the toxicity, whilst in current practice, mCRPC patients receive multiple cycle of [^177^Lu]Lu-PSMA-617 with limited toxicities. The study by Rahbar et al. showed that response (PSA reduction ≥ 50%) is only or mostly seen after the second cycle of [^177^Lu]Lu-PSMA-617 in men with mCRPC [9]. As this is a phase I study concerning treatment naïve prostate cancer patients with less tumour load, we think that one cycle of [^177^Lu]Lu-PSMA-617 is well grounded and can give additional anti-tumour effect. From an oncological point of view, adding additional treatment cycles will probably increase efficacy, without adding additional toxicity (based on salvage CRPC data). Upon successful dose-finding, adding cycles may be explored in subsequent research.

## Conclusion

The hypothesis of this study is that complementing EBRT and ADT with a single cycle concurrent [^177^Lu]Lu-PSMA-617 is safe, while providing synergistic anti-tumour effects with limited toxicity and without prolonging overall treatment time, with potential to improve tumour control and quality of life for future patients with N1M0 prostate cancer. In this phase I study, we aim to explore the tolerability of EBRT and ADT treatment combined with one cycle of [^177^Lu]Lu-PSMA-617.

## Supplementary Information

**Additional file 1: Table S1.** Overview of study logistics.

## Data Availability

The datasets generated during and/or analysed during the current study are available from the corresponding author on reasonable request.

## Abbreviations

α/β: Alpha/beta, fractionation sensitivity
ADT: Androgen DeprivationTtherapy
BOIN: Bayesian optimal interval
CCMO: Central committee on research involving human subjects; in Dutch: Centrale commissie mensgebonden onderzoek
CRF: Case report form
CT: Computed tomography
CTCAE: Common toxicity criteria adverse events
DLT: Dose limiting toxicity data safety monitoring board
eCRFs: Electronic case record forms European Organisation for research and treatment of cancer
EORTC: European Organisation for research and treatment of Cancer
EQD: Equivalent dose
GBq: Giga becquerel
GCP: Good clinical practice
GDPR: General data protection regulation; in Dutch: Algemene verordening gegevensbescherming (AVG)
GI: Gastro-intestinal
GU: Genito-urologic
Gy: Gray
IC: Informed consent
ICH: International Council on Harmonisation Investigational medicinal product
IMRT: Intensity modulated radiotherapy
IV: Intravenous
KeV: Kilo electron-volt
LND: Lymph node dissection
[^177^Lu]Lu-PSMA: [^177^Lu]Lutetium-177 labeled PSMA-ligands
mCRPC: Metastatic castration-resistant prostate cancer
METC: Medical research ethics committee (MREC); in Dutch: medisch-ethische toetsingscommissie (METC)
MRI: Magnetic resonance imaging
MTD: Maximum tolerated dose
NKI: Nederlands Kanker Intituut
NM: Nuclear medicine
PET: Positron emission tomography
PK: Pharmcokinectics
PSA: Prostate-specific antigen
PSMA: Prostate specific membrane antigen
QoL: Quality of life
RTOG: Radiation therapy oncology group
SBRT: Stereotactic body radiotherapy
SNP: Sentinel node procedure
SPECT: Single photon emission computed tomography
SV: Sievert
UMCU: University Medical Hospital Utrecht
WHO: World Health Organization
WMO: Medical research involving human subjects act; in Dutch: Wet medisch-wetenschappelijk onderzoek met mensen
