# Supplementary material for: Tolerability of concurrent external beam radiotherapy and [177Lu]Lu-PSMA-617 for node-positive prostate cancer in treatment naïve patients, phase I study (PROQURE-I trial)
Source: BMC Cancer. 2023 Mar 23;23:268. doi: 10.1186/s12885-023-10725-5 (PMC10035228; doi:10.1186/s12885-023-10725-5)
Supplement: Supplementary file 1 — Additional file 1: Table S1. Overview of study logistics. [file 12885_2023_10725_MOESM1_ESM.docx]

# Supplemental

# Table S1. Overview of study logistics

| **Treatment phase** | **Week** | **Standard of care** | **Study procedures** |
| --- | --- | --- | --- |
| Work-up |  | Standard work-up including PSMA PET/CT  Proven prostate cancer, staged iT2- 4N1M0 |  |
| Decision making |  | Multidisciplinary evaluation  Referred for EBRT prostate/nodes + 3 years ADT |  |
|  | -3 | Agree to standard treatment | Inclusion + informed consent  QoL questionnairesd |
|  |  | Initiation of ADT |  |
| Preparation | -2 | RT planning CT+MRI RT target delineation | Dose level selectiona Ordering [^177^Lu]Lu-PSMA-617 a  Logistical planninga |
|  | -1 | RT planning procedure |  |
| Treatment | 1 | EBRT + acute toxicity score CTCAEb |  |
|  | 2 | EBRT + acute toxicity score CTCAEb | 1x [^177^Lu]Lu-PSMA-617^a^ + 3x SPECT/CTc 11x blood samples for PKc |
|  | 3 | EBRT + acute toxicity score CTCAEb |  |
|  | 4 | EBRT + acute toxicity score CTCAEb |  |
|  | 5 | EBRT + acute toxicity score CTCAEb |  |
|  | 6 | EBRT + acute toxicity score CTCAEb |  |
|  | 7 | EBRT + acute toxicity score CTCAEb |  |
| Recovery | +1m |  | Acute toxicity score CTCAEb |
|  | +2m |  | Acute toxicity score CTCAEb |
| Follow-up | +3m | Clinical evaluation + PSA Acute toxicity score CTCAEb | QoL questionnairesd |
|  | +6m | Clinical evaluation + PSA Late toxicity score CTCAEb | QoL questionnairesd |
|  |  | Study ended. Continued FU according to standard of care |  |

Legend: Purple = treatment. Green = additional study procedures. ADT = Androgen deprivation therapy, CT = Computed Tomography, CTCAE = Common Terminology Criteria for Adverse Events, EBRT = External beam radiation therapy, FU = Follow-up, MRI = Magnetic resonance imaging, PET= Positron emission tomography, PK = pharmacokinetics, PSA = Prostate-specific antigen, PSMA = Prostate specific membrane antigen, QoL = Quality of life, RT = Radiotherapy, SPECT = Single positron emission tomography.

See below the table for additional details for specific components:

a: [^177^Lu]Lu-PSMA-617 therapy details: Single administered activity of 3, 6 or 9 GBq according to dose escalation step. Radiation safety measures for about two weeks during continued EBRT and at home.

b: Toxicity evaluation details: Acute and late toxicity is evaluated using CTCAE v. 5.0 criteria. When significant toxicity (grade three or higher) occurs, this may be evaluated with additional investigations as deemed necessary by the treating physician.

c: Biodistribution details: 3 x biodistribution SPECT/CT of groins-head at 4 hours, 1, 7 days after administration. 11 x blood samples for pharmacokinetics (PK) at pre-dose and at 5, 15, 30 minutes; 1, 2, 3, 6 hours; 1, 2-4, 7 days after administration.

d: Quality of life details: QLQ-C30 (general quality of life in oncology) and QLQ-PR25 (prostate-specific), at baseline and at three and six months after EBRT.
